# Investigation of Mechanical and Surface Properties of 3D-Printed Parts Aged Under Different Conditions (Coolant and Xenon Arc)

**DOI:** 10.3390/polym18182207

**Published:** 2026-09-10

**Authors:** Oğuz Koçar, Nergizhan Anaç, Erhan Baysal

**Affiliations:** 1 Department of Mechanical Engineering, Faculty of Engineering, Zonguldak Bülent Ecevit University, Zonguldak 67100, Türkiye; nergizhan.kavak@beun.edu.tr; 2 Alaplı Vocational School, Zonguldak Bülent Ecevit University, Zonguldak 67850, Türkiye; erhanbaysal@beun.edu.tr

**Keywords:** additive manufacturing, 3D printing, accelerated aging, xenon arc, coolant exposure, mechanical properties, optical properties

## Abstract

Predicting the changes in the performance of polymer-based 3D printing materials over time under industrial operating conditions is of critical importance. In particular, the chemicals present in the environments to which these materials are exposed and the duration of exposure directly affect their structural integrity and durability. In this study, the structural changes occurring in 3D-printed PLA Pro material as a result of aging in different environments (three different coolants used in the manufacturing industry and Xenon arc exposure) were investigated. The experiments were conducted using various analysis methods, including tensile and flexural tests, hardness, gloss, surface roughness, color, and mass measurements. The initial mass uptake behavior during the first 24 h of immersion was also evaluated. Finally, the changes in tensile strength (UTS) and flexural force of 3D-printed PLA Pro samples exposed to three different coolants for four different durations, and also subjected to 600 h of Xenon Arc aging, were evaluated using ANOVA. As a result, changes in the mechanical properties were observed under the different aging conditions. Compared with the reference specimen (41.9 MPa), the highest tensile strength was obtained for the specimens immersed in the water-based fully synthetic Rhenus TY108S coolant for two weeks (48.4 MPa), while the lowest tensile strength was observed for the specimens immersed in the semi-synthetic FU51 coolant for one week (39.9 MPa). Following Xenon arc aging, the tensile strength was determined to be 47.5 MPa. Regarding the maximum flexural force results, an increase was observed in the specimens aged under Xenon arc exposure, whereas a decrease was observed in the specimens aged in the coolants. Hardness generally decreased with increasing immersion time in the coolants; however, the hardness values remained higher than that of the reference PLA Pro specimen throughout the aging period. The highest color change occurred in the Xenon arc-aged specimens, while no significant color change was observed in the specimens exposed to the coolants. The surface gloss of all specimens was classified as matte to semi-matte. Surface roughness increased following exposure to the coolants, whereas it decreased after Xenon arc aging. The mass of the specimens was more strongly affected by coolant exposure.

## 1. Introduction

With advances in technology, changes in manufacturing methods continue to rapidly expand the application areas of additive manufacturing. Initially used for prototyping, additive manufacturing technologies are now employed in the production of final products, particularly offering high design flexibility and ease of manufacturing for complex geometries that are difficult to produce using conventional manufacturing methods. In 3D printing technologies, metal, ceramic, and polymer-based additive manufacturing techniques are used for production [1,2]. Among these techniques, Fused Deposition Modeling (FDM) is one of the most widely used methods due to its low cost, ease of application, and compatibility with a wide range of engineering materials [3,4]. The safe use of products obtained through the 3D printing process varies depending on the environmental conditions to which they are exposed. To understand the mechanisms underlying the effects of these conditions, researchers employ natural or artificial aging methods [5,6,7,8,9,10]. Osadolor et al. investigated the effects of UV aging on the mechanical properties of PLA (polylactic acid) structures and reported changes in specimens exposed to a temperature of 45 °C and a relative humidity of 65% for 4, 6, and 8 weeks. With increasing exposure time, the material was observed to lose its flexibility, while its elastic modulus and compressive strength increased [11].

Koçar et al. subjected PLA specimens with different colors (red, yellow, and orange) and infill ratios (20%, 60%, and 100%) to aging for 432 h at 50 °C and 50% relative humidity. Following the aging process, an increase in flexural force was observed in the specimens with a 100% infill ratio [12]. In another study, drone arms were fabricated using PLA reinforced with different additives. The fabricated specimens were subjected to artificial aging for 45 and 90 days. A decreasing trend was observed in the flexural test results during the first 45 days, whereas improvements in both tensile and flexural properties were observed after 90 days of aging [13]. Overall, previous studies provide information on changes in the mechanical properties of polymer specimens under exposure to UV radiation, temperature, salt spray, and humidity conditions. However, 3D-printed products used as engineering components are exposed not only to atmospheric conditions but also extensively to liquids, as they are used in mechanical manufacturing lines, moving mechanisms, and fixtures. As is well known, cooling and cutting fluids are widely used in industrial applications. Although the aging mechanisms resulting from the exposure of polymers to chemical liquids, acids, or oils have been extensively investigated in the literature, only a limited number of studies have examined how liquid absorption through the interlayer gaps and microporesof 3D-printed products affects their mechanical properties. In a recent study [14], the tensile and impact strengths of eight different commercial FDM filaments were compared after exposure to a chemical environment for 1 h, 24 h, and 7 days. These filaments were ABS (acrylonitrile butadiene styrene), ASA (acrylonitrile styrene acrylate), PA (polyamide), PC (polycarbonate), PETG (polyethylene terephthalate glycol), PLA, PP (polypropylene), and PVB (polyvinyl butyral). In the study, PP filament exhibited the highest chemical resistance, retaining more than 97% of its mechanical properties even in aggressive environments. In contrast, PLA, due to its low crystallinity and the presence of polar ester groups in its main chain, and PVB, due to its high amorphous content, showed the most pronounced degradation, particularly when exposed to acetone. Following acetone exposure, the loss in tensile strength of these two materials exceeded 70%, while their impact strength decreased to below 20%. In another study [15], the mechanical behavior of carbon/aramid hybrid-reinforced Elium composites manufactured using the hand layup-assisted vacuum infusion method was investigated after aging in pure water for up to six months. Tensile and flexural tests were performed on unaged specimens and specimens aged for 2, 4, and 6 months. It was reported that water absorption approached saturation after approximately two months and that the most pronounced changes in mechanical properties occurred during this period. After six months of aging, tensile strength decreased by 4.4%, while flexural strength decreased by 25.9%. Similarly, a study investigating the seven-day behavior of FDM-manufactured polyethylene terephthalate glycol-modified (PETG) and carbon fiber-reinforced PETG (PETG + CF) specimens exposed to engine oil (SAE 15W-40) reported that infill geometry and porosity, together with liquid absorption, directly affected mechanical performance [16]. While strength losses of up to 17% were observed in pure PETG, the carbon fiber-reinforced structure remained more stable under oil exposure. In light of these findings, there is a knowledge gap regarding the degradation of variants such as PLA Pro in specific chemical environments. Furthermore, how these industrial fluids interact with the porous interlayer gaps characteristic of FDM printing has not yet been investigated.

To address this gap, this study investigated the long-term behavior of PLA Pro specimens produced using FDM technology in both industrial liquid environments and a controlled artificial aging chamber, as well as the effects of these processes on the final performance of the material, was investigated. In the manufacturing industry, 3D printers have increasingly been used to produce fixtures. These fixtures are exposed to coolants during machining operations such as cutting, milling, and turning. This study has two main objectives. The first is to investigate the effects of coolant exposure on the mechanical properties of 3D-printed parts. The second is to compare the effects of coolant exposure and Xenon arc aging. Within the scope of this study, three different industrial coolants (TY108S, FU51, and TU426), which are widely used in the machining industry, were selected, and the specimens were subjected to controlled aging in these liquid environments for specified periods. Simultaneously, accelerated aging tests were conducted in a Xenon arc aging chamber to determine the material’s resistance to outdoor and atmospheric conditions. This approach provides new insights into whether FDM PLA Pro degradation is driven primarily by liquid absorption (chemical degradation) or by photo-oxidative degradation from atmospheric factors. Following the controlled aging processes, a comprehensive suite of characterization tests including tensile, three-point bending, hardness, mass, color change, surface roughness, and gloss measurements was conducted. Following the aging processes, tensile and three-point bending tests, hardness, mass, and color measurements, and surface roughness and gloss tests were performed to determine changes in the mechanical and surface characteristics of the material. Accordingly, the effects of both industrial coolants and controlled accelerated atmospheric aging conditions on the material were experimentally compared. In conclusion, this study provides a better understanding of the durability of PLA Pro, offering practical engineering knowledge for the use of FDM-printed components in industrial production environments.

In addition, using two-way and one-way ANOVA together with post hoc Tukey HSD tests, the present study statistically evaluates the changes in tensile strength (UTS) and flexural force of FDM-printed PLA Pro specimens exposed to three different coolants (TY108S, FU51, TU426) for four different durations (7, 14, 21, and 28 days), as well as to 600 h of Xenon Arc aging, relative to unexposed reference specimens.

## 2. Material and Method

**Figure 1:**
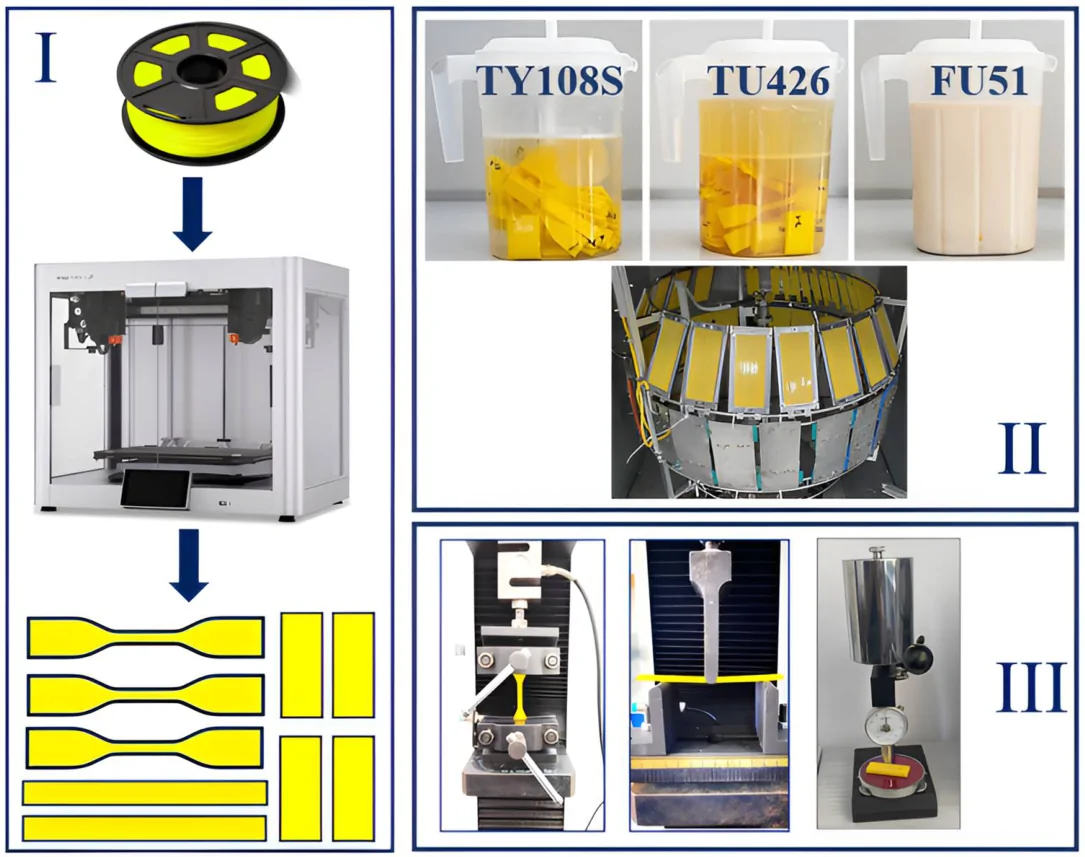
A flowchart of the study: (**I**) Printing of specimens using a 3D printer; (**II**) aging of specimens under Xenon Arc exposure and in coolants; (**III**) mechanical and optical tests.

The flowchart of the experimental study is presented in Figure 1. First, test specimens were printed using PLA Pro filament through the additive manufacturing process (Figure 1I). The fabricated specimens were then subjected to a controlled aging process for specified periods (Figure 1II). In the final stage, tensile, three-point bending, hardness, mass, color, surface roughness, and gloss tests were performed to investigate changes in the mechanical and optical properties of the specimens after aging (Figure 1III). For each experimental condition (tensile and flexural), four independent specimens (*n* = 4) were tested, and the average of the obtained results was calculated. The results were reported as the mean ± standard deviation.

### 2.1. Filament Properties

In the experiments, yellow polylactic acid-based PLA Pro filament manufactured by Elas (Singapore) was used. Due to its inherent characteristics, PLA is one of the most widely preferred printing materials in additive manufacturing, as it is non-toxic, has a high bio-based content, and is considered safe for human health. Owing to its organic sources, such as sugarcane and corn starch, it is also frequently used in environmentally friendly applications. The PLA Pro selected in this study is a modified and reinforced form of standard PLA and exhibits higher impact resistance and toughness than conventional PLA [17]. Since filament color and the pigments used are known to directly affect the mechanical properties of the material, all specimens were produced using the same yellow filament to reduce experimental variability and ensure result the consistency [5].

### 2.2. Experimental Design

To investigate the strength of specimens produced by additive manufacturing under industrial and environmental conditions, an experimental design comprising two different exposure groups was employed (Table 1). In the first experimental group, three different commercial coolants (TY108S, FU51, and TU426) (Manufacturer: Rhenus, Mönchengladbach, Germany; Supplier: Boğaziçi Industrial, İstanbul, Türkiye) were selected to determine the material behavior following exposure to industrial operating conditions. The specimens were immersed in these coolants for specified periods of 1, 2, 3, and 4 weeks. To ensure homogeneous contact between the liquids and the specimens, the liquids were stirred regularly each day. The immersion temperature of the samples in the coolant was 25.1 °C. Throughout the experiment, the concentration of the cooling fluids was monitored using a refractometer. The water/coolant ratio was kept constant throughout the experiment. The specimens were kept in a temperature- and light-controlled environment. At the end of each period (1, 2, 3, and 4 weeks), the PLA Pro specimens were removed from the cooling solution and gently dried with a clean, lint-free cloth to remove any excess solution remaining on the specimen surface prior to characterization and mechanical testing. The pH values of the cooling fluids were measured as 8.9 for FU51, 9.52 for TU426, and 9.6 for TY108S. The pH values were measured every 24 h over 28day period. At the end of the 28-day period, the pH values were determined to be 9.04 ± 0.1 for FU51, 9.52 ± 0.05 for TU426, and 9.63 ± 0.09 for TY108S. This indicates that there were no significant changes in the pH values of the cooling fluids during the experiment. The surface area of each sample was calculated, and the sample groups were placed in separate containers. The specimen-to-cooling fluid volume ratio was calculated as 5 mL/cm^2^. In the second experimental group, the specimens were subjected to artificial aging for 600 h using an Atlas Ci4000 Weather-Ometer (AMETEK CTS, Chicago, IL, USA) to investigate the effects of environmental conditions. To clearly compare the effects of the aging processes on the tensile, flexural, hardness, mass, surface roughness, color, and gloss properties of the specimens, the test results were compared with those of the untreated specimens.

### 2.3. Properties of Coolants

Three commercial coolants with different chemical compositions were selected for use in the aging experiments on PLA Pro specimens produced via the FDM method. These coolants provide a range of chemical compositions, from synthetic to emulsion-based formulations, with varying water and mineral oil contents, allowing for a comparative investigation of the behavior of PLA Pro under different liquid environments [18,19]. In this context, Rhenus TY108S, selected as the first aging medium, is a fully synthetic, water-based grinding fluid containing no mineral oil [20]. This fluid, which contains organic and inorganic corrosion inhibitors, was included in the experimental process because it represents an environment in which hydrolytic aging effects on the PLA matrix may be more pronounced [21]. Rhenus FU 51, selected as the second aging medium, has a semi-synthetic micro-emulsion formulation containing both water and mineral oil phases [22]. This characteristic provides an intermediate exposure environment in which polar and less polar components coexist, allowing the liquid absorption behavior of PLA Pro specimens to be evaluated under more complex conditions [23]. The third coolant used in the experimental process, Rhenus TU 426, is a cutting fluid with a high mineral oil content that forms an emulsion when mixed with water [24]. This fluid was selected to represent an aging environment in which the effect of the hydrophobic phase is more pronounced. Thus, the experimental process enabled a comparative aging assessment across water-based, semi-synthetic, and oil-rich environments [25,26]. To represent industrial operating conditions, all coolant concentrates were diluted with pure water to a volumetric concentration of 5% (5% *v*/*v*).

### 2.4. Aging Experimental Setup

The accelerated aging tests in this study were conducted using a Xenon arc lamp in accordance with EN ISO 4892-2:2013 [27], which specifies standards for laboratory light sources. Method A (Cycle No. 1), defined under the “Xenon-arc lamps” section of the standard, was followed. An Atlas Ci4000 Weather-Ometer was used for the aging tests, with a Type S boro/Type S boro combination used for the inner and outer filters. Throughout the test cycle, the specimens were exposed to a constant irradiance of 0.51 W/(m^2^·nm) at a wavelength of 340 nm. The cycle consisted of a continuous 102 min dry phase followed by an 18 min wet (spray) phase. During the dry phase, the black standard thermometer (BST) temperature was maintained at 65 ± 3 °C, the chamber temperature at 38 ± 3 °C, and the relative humidity at 50 ± 10%. The test specimens were subjected to an exposure period of 600 h. To minimize regional variations arising from the distribution of light, temperature, and humidity within the chamber and to maximize the repeatability of the test results, the specimen rack was continuously rotated at a speed of 1 rpm.

### 2.5. Three-Dimensional Printing Parameters and Tests

**Figure 2:**
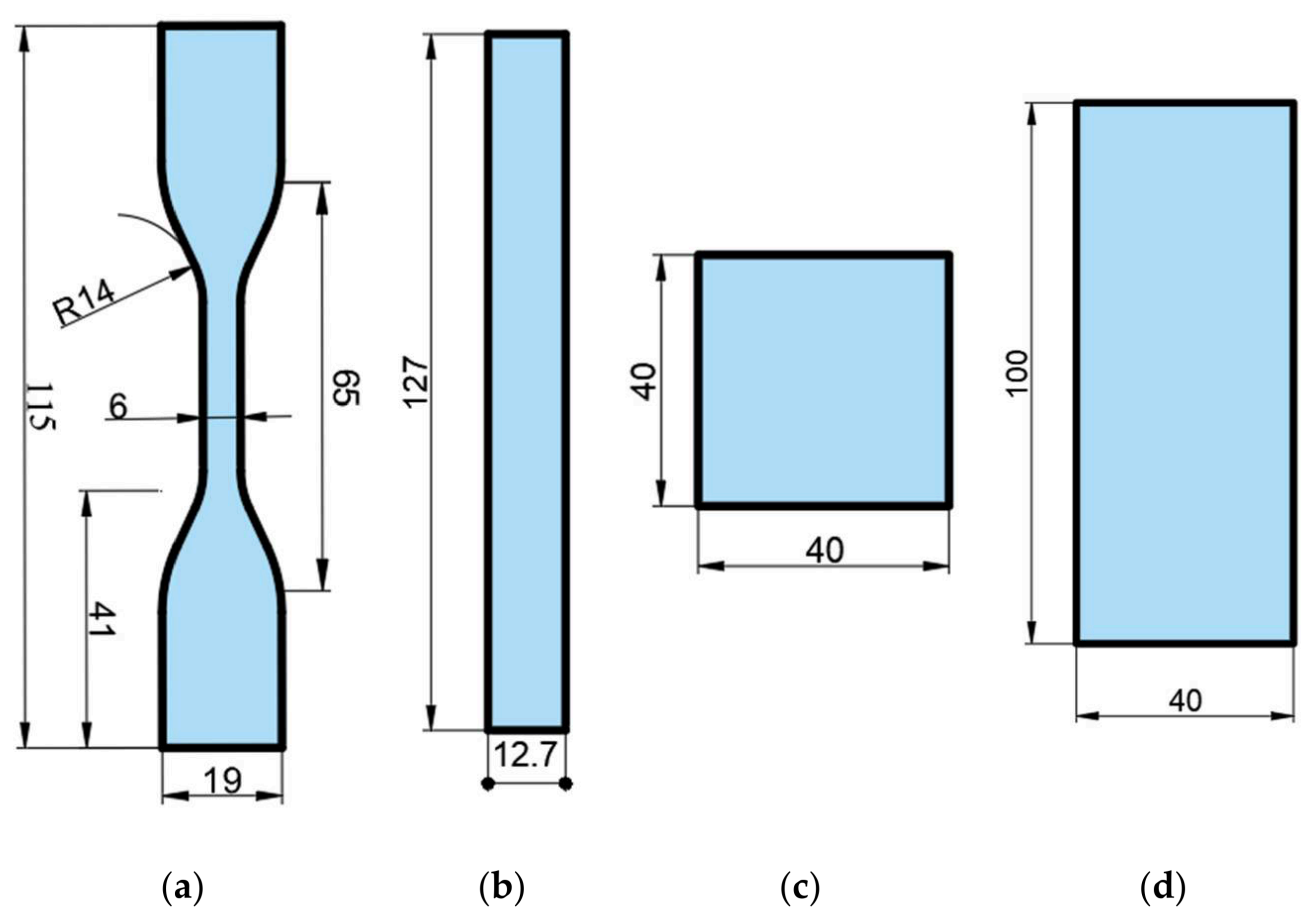
Dimensions of specimens used for mechanical tests: (**a**) tensile test, (**b**) flexural test, (**c**) hardness test, and (**d**) color, surface roughness, and gloss tests (dimensions are in mm).

The additive manufacturing process for all specimens was carried out using a Snapmaker J1s 3D printer (Shenzhen Snapmaker Technologies Co., Ltd., Shenzhen, China) equipped with an independent dual-extruder system. The slicing process was performed using Snapmaker Orca slicer 2.3.0 software. Prior to printing, the PLA Pro filaments were used as received without any additional drying procedure. To ensure that the fabricated specimens had identical characteristics and consistent quality, the printing parameters were strictly controlled. PLA is a hygroscopic (moisture-absorbing) material. To prevent any adverse effects resulting from this property of PLA, the PLA Pro filament used in the study was used for production immediately upon removal from its vacuum-sealed package. Pre-drying was not applied. The printing of all specimens was completed continuously without interruption to the 3D printing process. During printing, no flow instability was encountered at the nozzle, and no clogging occurred. No brittleness issues were observed in the filaments. A 0.4 mm diameter brass nozzle was used with an extrusion width of 0.4 mm. The infill ratio was set to 100% with a linear pattern; however, micro voids may still inherently exist within the printed structure. All specimens were printed in the flat/XY build orientation on the build plate. The printing speed was set to 60 mm/s, the nozzle temperature to 210 °C, and the build plate temperature to 60 °C. The layer thickness was specified as 0.2 mm. Furthermore, the raster orientation was set to ±45°, and the structure was fabricated with 3 walls/shells and 3 top/bottom solid layers. Standard-compliant specimens were used for mechanical characterization tests conducted in the experimental study. Specimens with geometries conforming to ASTM D638 (Type IV) [28] were produced for tensile strength testing (Figure 2a), while three-point bending specimens conforming to ASTM D790 [29] were produced to determine the flexural properties (Figure 2b).

The mechanical tests were performed using a WDW-5 universal testing machine equipped with a 5 kN load cell. For the tensile tests, the crosshead speed was set to 5 mm/min, and the tensile strain was recorded using the crosshead displacement. For the three-point bending tests, the crosshead speed was also set to 5 mm/min, and the support span length was adjusted to 56 mm, yielding a span-to-depth ratio of 16:1 in accordance with the standard. All mechanical tests were conducted under controlled environmental conditions at room temperature. As stated in the experimental design, four independent specimens were tested for each experimental condition to evaluate repeatability, and the average values were reported.

The hardness values of the specimens were determined in accordance with ASTM D2240 [30]. To ensure the accuracy and repeatability of the hardness data, measurements were taken at five different points on the surface of each specimen, and the arithmetic mean of these values was calculated. The dimensions of the specimens used for the Shore D hardness test are given in Figure 2c. Figure 2d shows the dimensions of the specimens used for the color, surface roughness, and gloss tests. The mass of the specimens was measured using a precision balance. A PCE-CSM 25 (PCE Instruments, Meschede, Germany) color measuring device was used to determine color changes, while a PCE-PGM 100 gloss meter (PCE Instruments, Meschede, Germany) was used for surface gloss measurements. Surface roughness was measured on the upper surface of the specimens using a Mitutoyo SJ301 portable surface roughness tester (Mitutoyo Corporation, Kawasaki, Japan). The average arithmetic surface roughness (Ra) and mean roughness depth (Rz) values were recorded for directions parallel to the tensile test direction. Five measurements were taken from each specimen, and the mean values were calculated. Five measurements were performed for each specimen, and the mean values were calculated. A sampling length (cut-off) of 0.8 mm and an evaluation length of 4 mm were used for the measurements.

To evaluate the effects of coolant type and exposure duration on the tensile strength (UTS) and flexural force of 3D-printed PLA Pro specimens, two-way Analysis of Variance (ANOVA) was performed separately for each mechanical property at a significance level of α = 0.05 [31]. Since only a single observation was available for each coolant × duration combination (*n* = 1/cell), the model was constructed as an additive main-effects model without an interaction term. When at least one main effect was found to be significant, post hoc Tukey HSD (Honestly Significant Difference) tests were used for pairwise group comparisons; for the five-group comparisons with unbalanced sample sizes (the reference and xenon groups had *n* = 1), the Tukey–Kramer correction was applied. In addition, a separate one-way ANOVA was performed to evaluate overall differences among all environmental exposure conditions (the unexposed reference and the 600 h xenon arc-aged group included five groups in total). Model assumptions were verified for both mechanical properties using Levene’s test (homogeneity of variance) and the Shapiro–Wilk test (normality of residuals). Effect size was reported using partial eta-squared (η^2^p).

### 2.6. Determination of Mass Change During Initial Immersion

To investigate the initial mass uptake behavior of the PLA Pro specimens, additional immersion experiments were conducted during the first 24 h. The specimens were immersed in TY108S, FU51, and TU426 cooling solutions, and their masses were measured at 0, 1, 2, 4, 8, 12, and 24 h. Four replicate specimens were evaluated for each coolant. At each measurement interval, the specimens were removed from the solution and gently dried using a clean, lint-free cloth to remove excess solution from the specimen surface before weighing. The mass change (%) was calculated according to Equation (1):

(1) $$Mass\;change\;(\%)=\frac{m_{t}-m_{0}}{m_{0}}\times 100$$

where $m_{0}$ represents the initial mass of the specimen and $m_{t}$ represents the mass after the corresponding immersion time.

## 3. Results and Discussion

### 3.1. Tensile Test Results

**Figure 3:**
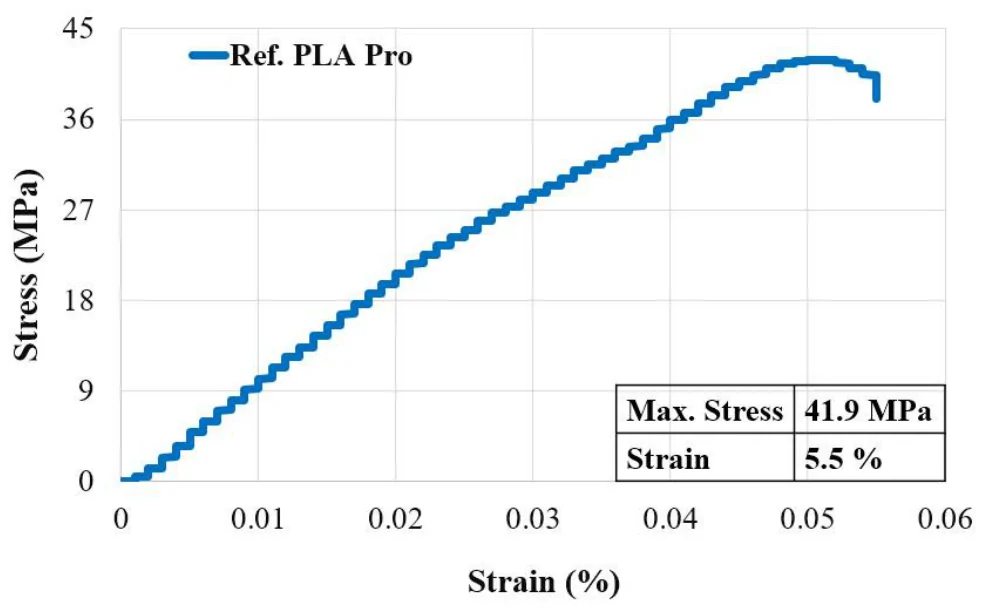
Stress–strain curve of reference material.

Figure 3 presents the stress–strain curve of the reference PLA Pro specimen. The maximum tensile stress was measured at 41.9 ± 1.9 MPa, with a corresponding strain of 5.5 ± 0.7%. Özkara et al. investigated the mechanical properties of eight different eSUN PLA Plus filaments. They reported that the highest tensile strength was 59.06 MPa for the white filament, the lowest was 49.94 MPa for the blue filament, and the tensile strength of the yellow was 54.32 MPa [32]. Parmaksız et al. investigated the effects of layer thickness on the mechanical and thermal properties of PLA (Ultimaker), PLA Plus (eSUN), PLA CF (Filameon), PLA Wood (Filameon), Tough PLA (Ultimaker), ABS+ (eSUN), and TPU 92A (Sava) filaments. At a layer thickness of 0.2 mm, the tensile strengths were determined as 54.41 MPa for PLA, 49.31 MPa for PLA Plus, 54.08 MPa for PLA CF, 36.55 MPa for PLA Wood, 51.33 MPa for Tough PLA, 31.85 MPa for ABS+, and 36.45 MPa for TPU 92A [33]. These results indicate that the mechanical properties of filaments used in 3D-printed filaments may vary depending on the manufacturer and printing parameters.

**Figure 4:**
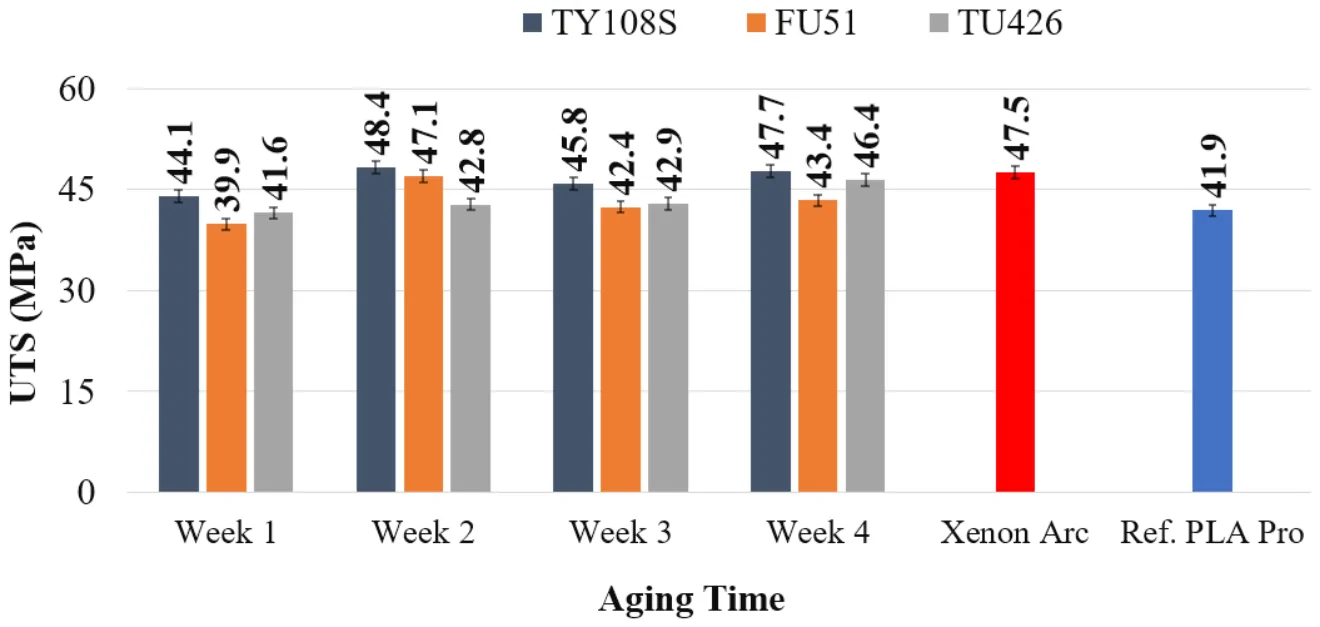
Comparison of the tensile strengths of the specimens.

Figure 4 presents the ultimate tensile strength (UTS) values of all specimens after exposure to different aging environments and durations, in comparison with the reference group. Table 2 presents the stress–strain values of the specimens exposed to the coolants. Table 3 presents the two-way ANOVA summary for the UTS values obtained across the three coolants (TY108S, FU51, TU426) and four exposure durations (7, 14, 21, and 28 days). The main effect of coolant type on UTS was statistically significant (F(2, 6) = 5.31; *p* = 0.047; partial η^2^ = 0.64), indicating that coolant selection was significantly associated with the tensile performance of the PLA Pro specimens. In contrast, the main effect of exposure duration was not statistically significant at the 5% level (F(3, 6) = 4.62; *p* = 0.053; partial η^2^ = 0.70); accordingly, no post hoc comparisons were performed for exposure duration, and the corresponding findings are reported only as numerical trends. The model explains a large proportion of the total variance (R^2^ = 80.32%; adjusted R^2^ = 63.91%), indicating that the two selected factors account for a substantial share of the variability in UTS.

The results show that the UTS values varied depending on the exposure environment and duration compared with the reference value of 41.9 MPa. The tensile strength of the PLA Prospecimens aged directly in the aging chamber without exposure to any liquid (Xenon arc) increased by 13.4 ± 1.1%, reaching 47.5 ± 2.1 MPa. When evaluated at weekly intervals, the tensile strength of the PLA Pro specimens exposed to TY108S remained above the reference value throughout all exposure periods. The highest tensile strength in this group was obtained at the end of the second week (48.4 MPa), while the lowest increase was recorded after the first week (44.1 MPa). In contrast, the tensile strengths of the specimens exposed to FU51 and TU426 were below that of the reference material only during the first week of aging, with values of 39.9 MPa and 41.6 MPa, respectively. During the subsequent weeks, an increasing trend relative to the reference value was observed for both coolant groups.

In a similar study in the literature, PLA specimens with different infill ratios (40%, 60%, 80%, and 100%) were exposed to coolant for 7 and 30 days. The researchers reported a tensile strength of 45 MPa and an elongation at break of 4.68% for the reference PLA specimen with a 100% infill ratio. Following coolant exposure, the tensile strength of these fully filled specimens (100% infill ratio) decreased to 39.47 MPa after 7 days, while elongation decreased to 4.26%. After 30 days, the tensile strength and elongation were reported as 38.65 MPa and 4.32%, respectively. The tensile strength values obtained for the specimens exposed to FU51 and TU426 at the end of the first week are consistent with those reported in the literature [34].

Post hoc Tukey HSD analysis showed that the TY108S coolant yielded the highest numerical mean UTS value (46.50 ± 1.94 MPa), followed by TU426 (43.43 ± 2.07 MPa) and FU51 (43.20 ± 2.99 MPa). Although TY108S exhibited the highest numerical mean UTS, none of the pairwise comparisons among coolants were statistically significant after Tukey HSD adjustment for multiple comparisons (Table 4). Therefore, although the overall main effect of coolant type was significant, the specific coolant pair responsible for this difference cannot be conclusively identified with the present sample size (*n* = 4/group); this is consistent with the finding of Turner et al. [35] that the effect of coolant chemistry on polymers is formulation-specific and typically characterized by overlapping confidence intervals.

Furthermore, when all exposure conditions (Reference: 41.90 MPa; 600 h of xenon arc aging: 47.50 MPa; and coolant groups) were compared together, a one-way ANOVA indicated that the differences among the five groups were not statistically significant (F(4, 9) = 1.90; *p* = 0.194) (Table 5). Therefore, the changes observed among the reference, Xenon arc-aged, and coolant-exposed samples were interpreted as numerical trends rather than statistically significant differences.

### 3.2. Flexural Test Results

**Figure 5:**
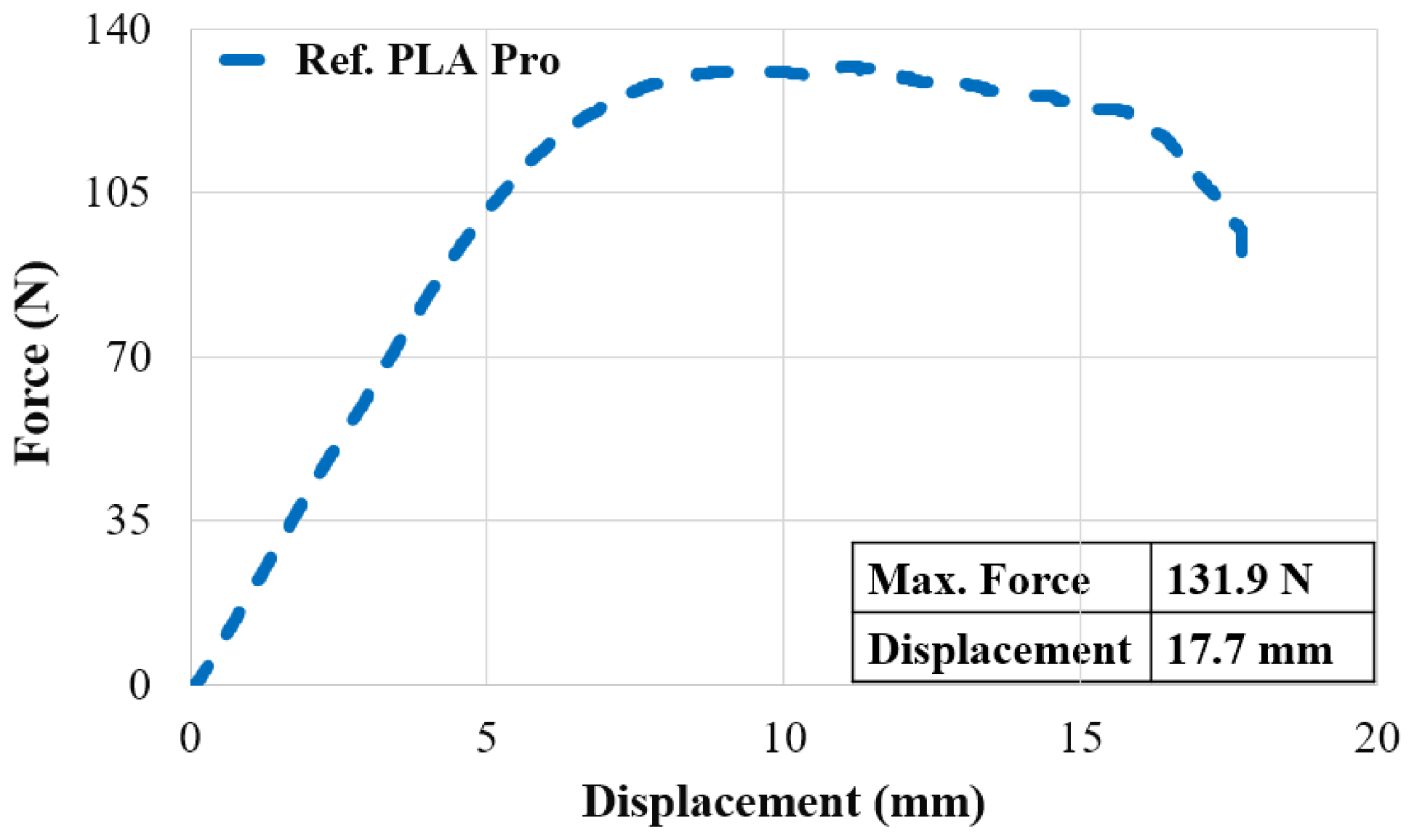
Force–displacement curve of reference material.

Figure 5 presents the force–displacement curve of the reference PLA Pro specimen. The maximum flexural force was measured at 131.9 ± 4.6 N, with a corresponding displacement of 17.7 ± 2.4 mm. Özkara et al. investigated the flexural force of eight different eSUN PLA Plus filaments. The highest flexural force was reported for the black filament (108.7 N), the lowest for the blue filament (89.4 N), while the value for the golden yellow filament was reported as 92.3 N [32].

**Figure 6:**
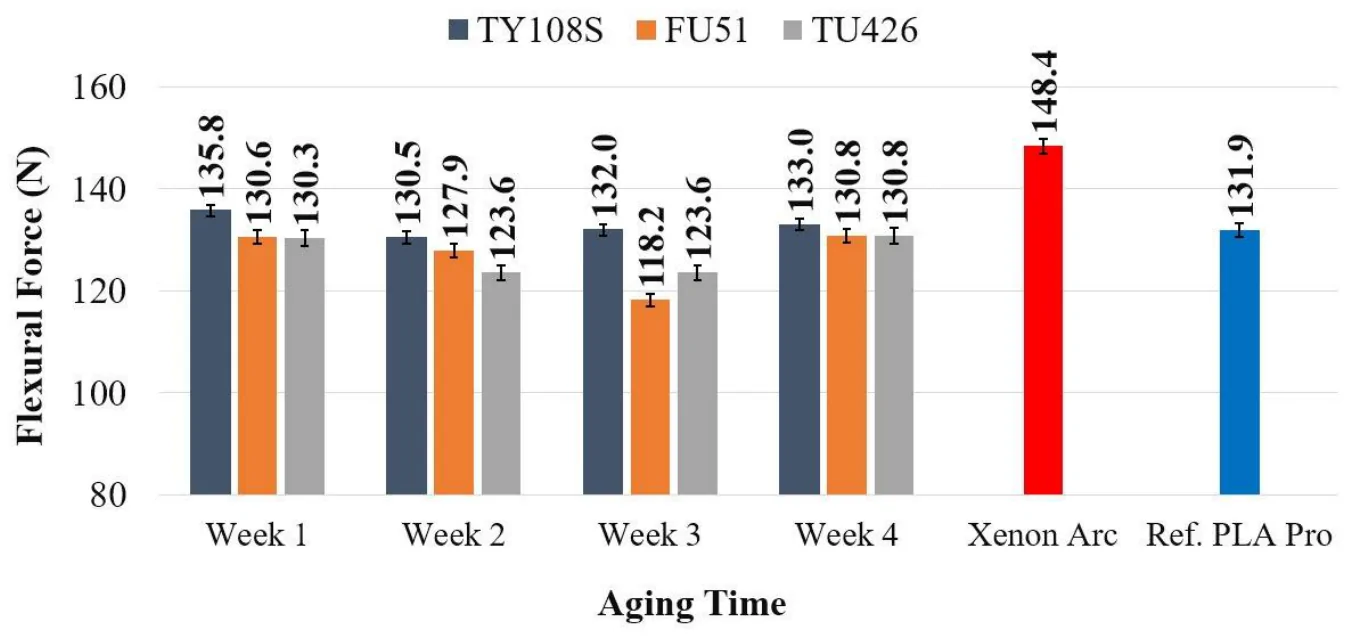
Flexural force of specimens.

Figure 6 presents the maximum flexural force values of all specimens after exposure to different aging environments for different durations in comparison with the reference group. Table 6 presents the force–displacement values of the specimens exposed to the coolants. The results show that, compared with the reference value of 131.9 N, the maximum flexural force of the PLA Pro specimens aged directly in the aging chamber without exposure to any liquid (Xenon arc) increased by 12.5 ± 1.2%, reaching 148.4 ± 2.2 N. When evaluated at weekly intervals, the maximum flexural force of the PLA Pro specimens exposed to TY108S remained above the reference value at Week 1 (135.8 N) and Week 4 (133.0 N). In contrast, the maximum flexural force of the specimens aged in FU51 and TU426 remained below that of the reference material. The lowest flexural force during the test period was recorded for the specimens exposed to FU51 at Week 3 (118.2 N).

The same statistical framework was subsequently applied to the flexural force data included in the dataset. Table 7 presents the two-way ANOVA results for flexural force across the three coolants and four exposure durations. Consistent with the UTS findings, the main effect of coolant type on flexural force was statistically significant (F(2, 6) = 5.27; *p* = 0.048; partial η^2^ = 0.64). The main effect of exposure duration was again at the margin of significance but did not reach the 5% level (F(3, 6) = 4.50; *p* = 0.056; partial η^2^ = 0.69). The model accounts for a large share of the variance in flexural force (R^2^ = 80.01%; adjusted R^2^ = 63.36%).

Post hoc Tukey HSD analysis showed that, as with UTS, the TY108S coolant yieldedthe highest numerical mean flexural force (132.83 ± 2.23 N), followed by TU426 (127.08 ± 4.02 N) and FU51 (126.88 ± 5.93 N). Consistent with the UTS finding, none of the pairwise coolant comparisons reached significance after family-wise error rate (FWER) correction (Table 8); this indicates that the effect of coolant type follows a consistent trend for both mechanical properties (TY108S > TU426 ≈ FU51), although the specific pairwise differences cannot be distinguished within the limits of the present sample size.

Unlike the UTS finding, the one-way ANOVA encompassing all exposure conditions (Reference, xenon, and the three coolant groups) revealed a statistically significant overall difference in flexural force (F(4, 9) = 5.90; *p* = 0.013) (Table 9).

To identify the source of this significant overall effect, a Tukey–Kramer post hoc test was applied (Table 10). The results showed that the flexural force of the specimens subjected to 600 h of xenon arc aging (148.4 N) was statistically significantly higher than that of both the FU51 (126.9 N; *p* = 0.011) and TU426 (127.1 N; *p* = 0.011) coolant groups. The differences between the Xenon Arc group and the TY108S (132.8 N; *p* = 0.062) and Reference (131.9 N; *p* = 0.133) groups were close to the significance threshold but were not statistically significant at the 5% level. This finding suggests that, at least at the 600 h exposure duration applied in this study, xenon arc aging—rather than reducing flexural rigidity—likely increased it, possibly because the photo-oxidative effect remained confined to the specimen surface while a secondary crystallization or post-cure-like stiffening effect occurred within the bulk material; this interpretation is consistent with the literature reporting that short-term UV/xenon exposure can increase modulus prior to the onset of chain scission [36].

### 3.3. Hardness Results

**Figure 7:**
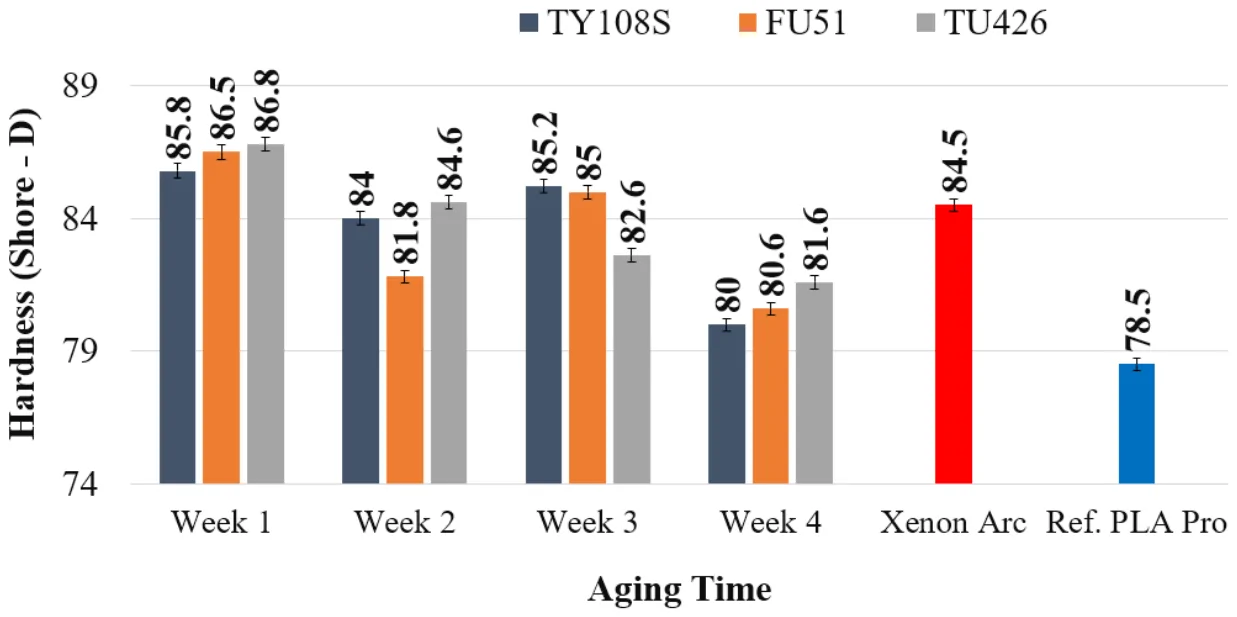
Hardness values of specimens.

Figure 7 presents the hardness values of all specimens after exposure to different aging environments and durations in comparison with the reference group. Overall, the hardness values of all specimens aged both in the coolants and under Xenon arc exposure showed a noticeable increase compared with the reference specimen. This increase may be attributed to thermal effects during the aging processes or to the post-crystallization mechanism induced by the liquid environments [37]. The highest hardness values in all environments were obtained at the end of Week 1. The highest value was recorded for TU426 at 86.8 Shore D, followed by FU51 (86.5 Shore D) and TY108S (85.8 Shore D). However, as the exposure period progressed toward Week 4, the hardness values gradually decreased due to continuous liquid diffusion into the polymer matrix, which weakened the interlayer bonds and caused a plasticization effect [38]. Accordingly, the hardness values decreased to 80.0, 80.6, and 81.6 Shore D. On the other hand, the hardness of the specimens subjected to controlled artificial aging in the Xenon arc chamber was measured at 84.5 Shore D. This relatively high and stable hardness, without the adverse effects of hydrolytic degradation and plasticization associated with liquid environments [39], was attributed to the increase in material crystallinity induced by UV radiation and continuous thermal cycling.

### 3.4. Initial Mass Change During the First 24 Hours and Mass Measurement Results

**Figure 8:**
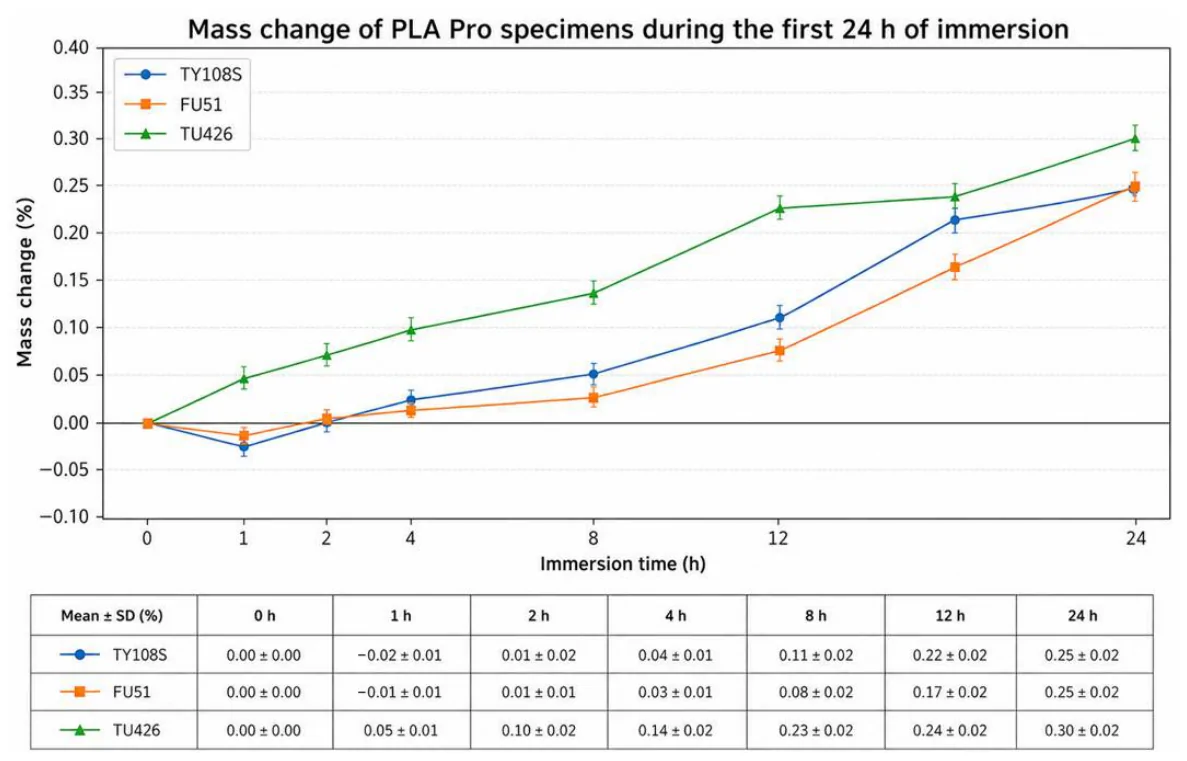
The mass change of the PLA Pro.

Figure 8 presents the mass change of the PLA Pro specimens during the first 24 h of immersion in the three cooling solutions. The specimens exhibited relatively limited mass changes throughout the initial immersion period. The TY108S specimens showed an initial decrease in mass at 1 h, followed by a gradual increase, reaching approximately 0.25% mass increase after 24 h. In comparison, the FU51 specimens exhibited a relatively gradual increase in mass during the immersion period, reaching approximately 0.25% at 24 h. The TU426 specimens showed the highest mass increase during the first 24 h, reaching approximately 0.30% at 24 h.

The relatively small mass changes observed during the first 24 h indicate that the initial interaction between the PLA Pro specimens and the cooling solutions was limited. The differences among the cooling solutions became more apparent during the early immersion period, particularly for TU426, which exhibited a faster increase in mass compared with TY108S and FU51. However, the mass change curves should be interpreted primarily as an indication of initial solution uptake rather than direct evidence of polymer swelling, since the measurements represent changes in specimen mass.

**Figure 9:**
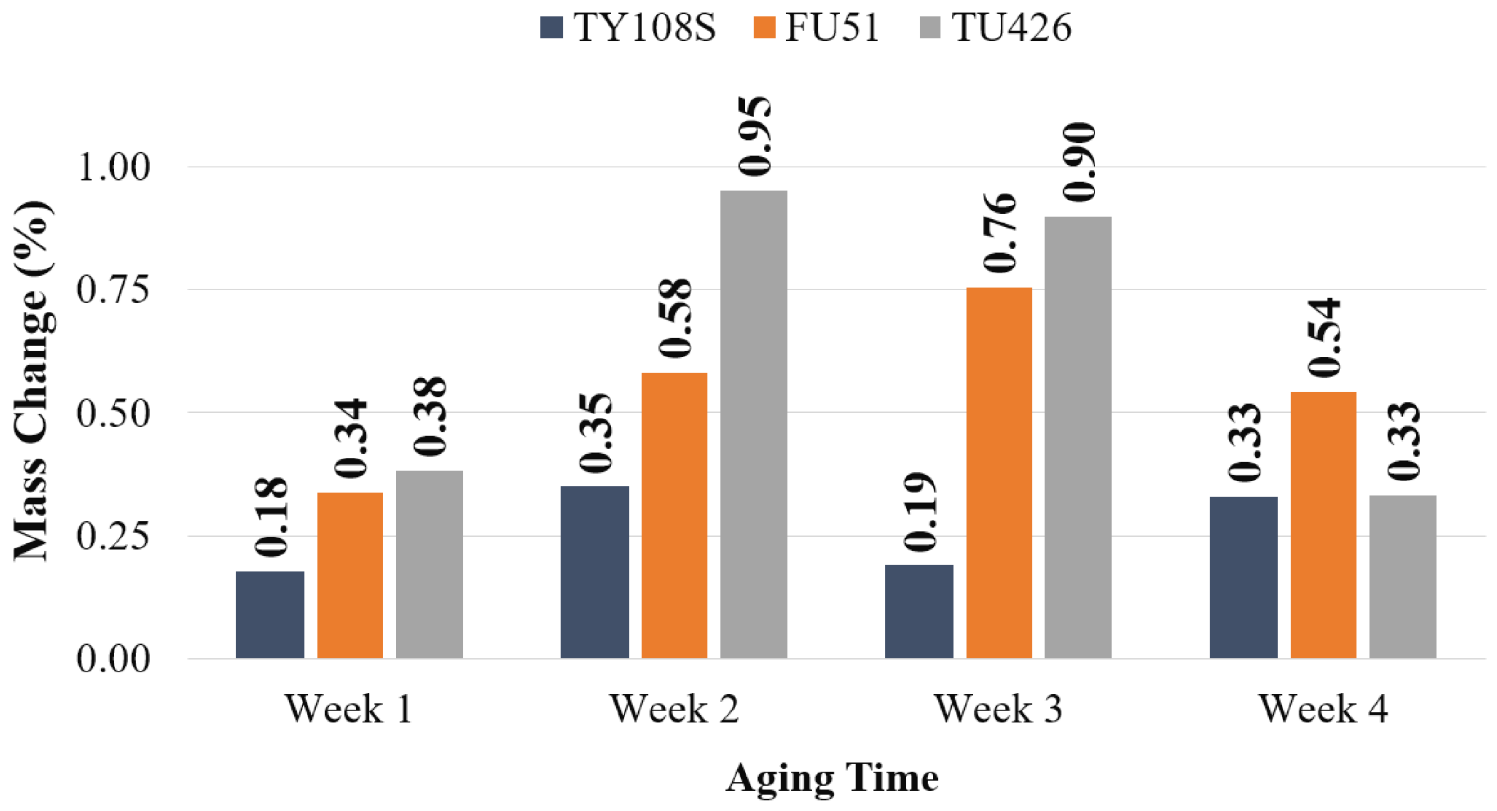
Mass change according to coolant type and exposure duration.

Figure 9 presents the mass change rates (%) of the specimens exposed to three different coolants (TY108S, FU51, and TU426) over the four-week aging period. The changes were calculated based on mass measurements performed before and after aging. TY108S exhibited the lowest mass increase throughout the test period (0.18% at Week 1 and 0.33% at Week 4), indicating the lowest level of liquid penetration into the specimens. In contrast, the highest liquid absorption was observed in the specimens exposed to TU426, reaching a maximum of 0.95% at Week 2 before decreasing sharply to 0.33% at the end of Week 4.

The specimens exposed to FU51 showed a gradual increase in mass during the first three weeks (0.34%, 0.58%, and 0.76%), followed by a decrease to 0.54% in the last week. The relatively high mass gain observed at Week 2 across the coolant groups, followed by a general decreasing trend toward the end of Week 4, can be explained by moisture diffusion [40]. Moisture diffusion is a phenomenon characterized by an increase in the mass of a material after exposure to a wet environment. The mass change trends of the PLA Pro materials are consistent with the results reported by Banjo et al. [41].

In contrast, the average mass change after Xenon arc aging was only −0.065%, indicating that the overall mass loss under these aging conditions was relatively limited.

### 3.5. Color and Gloss Measurement Results

The specimens were visually inspected before the color and gloss measurements. The color and brightness evaluations obtained in this study are specific to the yellow PLA Pro filament material only and should not be generalized to other colors. The measurements were conducted in accordance with ASTM D2244 [42], with three measurements taken per sample. Visual inspection revealed that the color of the Xenon arc-aged specimens differed from that of the other specimens. The CIE L*a*b* color system was used to evaluate the color of the PLA Pro specimens. The measurement values of the aged and reference (unaged) specimens are presented in Table 11. CIE L*a*b* is a three-dimensional international color system defined by the L* (lightness–darkness), a* (green–red), and b* (blue–yellow) coordinates. The equations used to calculate the total color change (ΔE*) between the aged and reference specimens are given in Equations (1)–(5).

(2) $$\begin{array}{c} ∆E^{*}=\sqrt{{\left(∆L^{*}\right)}^{2}+{\left(∆a^{*}\right)}^{2}+{\left(∆b^{*}\right)}^{2}}\;\;\;\;\; \end{array}$$

(3) $$\begin{array}{c} ∆E^{*}=\sqrt{{\left(L_{2}^{*}-L_{1}^{*}\right)}^{2}+{\left(a_{2}^{*}-a_{1}^{*}\right)}^{2}+{\left(b_{2}^{*}-b_{1}^{*}\right)}^{2}}\;\;\;\;\; \end{array}$$

(4) $$\begin{array}{c} ∆L^{*}=L_{2\left(aged\;specimen\right)}^{*}-L_{1\left(unaged\;specimen\right)}^{*}\;\;\;\;\; \end{array}$$

(5) $$\begin{array}{c} ∆a^{*}=a_{2\left(aged\;specimen\right)}^{*}-a_{1\left(unaged\;specimen\right)}^{*}\;\;\;\;\; \end{array}$$

(6) $$\begin{array}{c} ∆b^{*}=b_{2\left(aged\;specimen\right)}^{*}-b_{1\left(unaged\;\;specimen\right)}^{*}\;\;\;\;\; \end{array}$$

Compared with the reference specimens, the highest total color change was observed after Xenon arc aging. In contrast, the color changes in the specimens exposed to the coolants did not differ considerably from those of the reference specimens. Moreover, regardless of the type of coolant, the color changes measured in these specimens were similar. The value of ΔE* > 5.0 is perceived by the human eye as representing two completely different colors, and at this level, the part exceeds the limit of industrial acceptability [43]. The ΔE* value observed after xenon arc aging would not be considered acceptable for the commercial or functional usability of the part.

Differences were observed in the surface gloss of the specimens. In particular, the difference between the specimens exposed to the coolants and the other specimens was more pronounced. Gloss values (GU) were measured at angles of 20°, 60°, and 85°, and the results are presented in Table 12.

Gloss measurements were performed in accordance with ASTM D523 [44]. Gloss measurements were obtained from three readings per sample. The 60° gloss values of both the reference and Xenon arc-aged specimens were below 10 GU, indicating matte surfaces. Based on the 85° measurement angle, which is preferred for evaluating matte surfaces, the gloss of the reference specimens decreased from 15.3 GU to 12.2 GU after Xenon arc aging. A similar trend was observed for the TY108S (Week 4) and TU426 (Weeks 1 and 3) specimens exposed to the coolants; however, the surfaces of these specimens were more matte than those of the Xenon arc-aged specimens. The other specimens exposed to the coolants exhibited gloss values ranging from 10.1 to 11.3 GU and were classified as having semi-matte surfaces. Therefore, these surfaces exhibited a relatively glossier appearance. Table 13 compares the surface appearances of the reference and Xenon arc-aged specimens with those of the specimens immersed in TY108S, FU51, and TU426 coolants for 1–4 weeks. The specimens immersed in TY108S exhibited only limited color change. The L*, a*, and b* values were close to those of the reference specimen throughout the four-week period, indicating that this coolant had no considerable effect on the color appearance of the specimen surface. In particular, the change in the b* value from 79.35 to 79.97 indicates only a limited variation in the yellow color component. For the FU51 specimens, decreases in the L* and b* values were observed at Week 2, and then partially approached the reference levels in the subsequent weeks. This suggests that the color change exhibited a limited and fluctuating pattern with increasing immersion time.

For the TU426 specimens, the most notable change was observed at Week 3. The decrease in the L* value to 80.26 and the b* value to 77.98 indicates a noticeable reduction, particularly in the yellow color component. As can also be seen in the image, the Week 3 specimen has a different color tone compared with the other TU426 specimens. The b* value of the reference specimen was 79.23, whereas it decreased to 63.71 after Xenon arc aging. This indicates a much more pronounced color change than the relatively small changes observed following coolant exposure.

### 3.6. Surface Roughness Measurement Results

**Figure 10:**
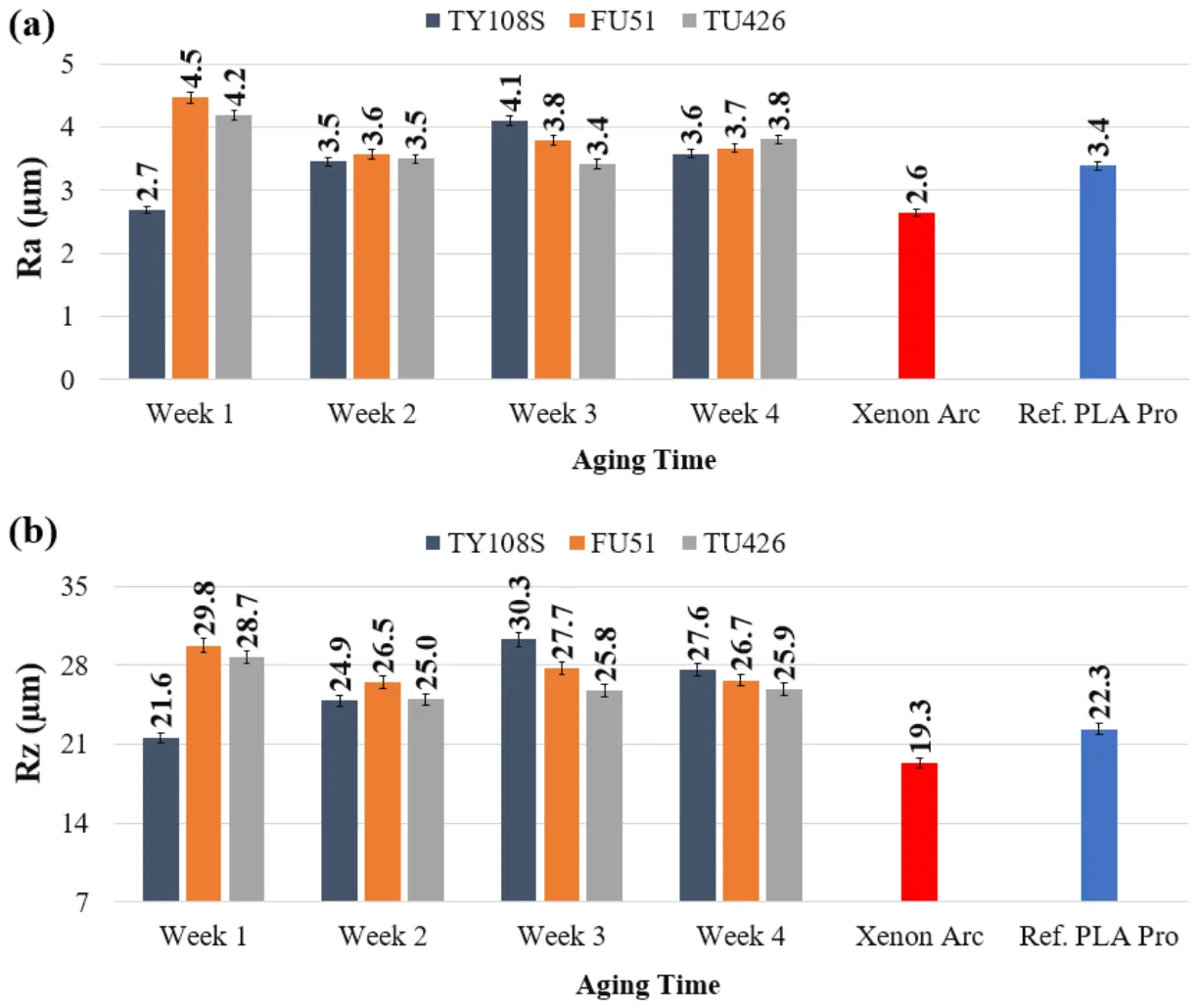
Surface roughness values of specimens: (**a**) Ra and (**b**) Rz.

The average arithmetic surface roughness (Ra) and mean roughness depth (Rz) values of the specimens are presented in Figure 10a and Figure 10b, respectively. The Ra value indicates the overall roughness of the surface. The Ra value of the reference PLA Pro specimens was measured at 3.4 µm, while that of the specimens after Xenon arc aging was 2.6 µm. It has also been reported in the literature that the surface roughness of polymers decreases after Xenon arc aging depending on the type of polymer [45]. In contrast, the Ra values of the specimens exposed to the coolants increased compared with that of the reference material. This indicates that the surface became rougher following exposure to the liquids. Rz (mean roughness depth) is a roughness parameter that represents the average distance between the highest peaks and the deepest valleys in the surface profile of the specimens. Rz is more sensitive than Ra in measuring sharp peaks or deep scratches. This parameter was measured because it indicates surface irregularities on the surfaces exposed to the coolants where the liquid may adhere or penetrate. The Rz graph showed a trend similar to that of the Ra values, except for the Week 4 values. For the specimens exposed to the liquids for four weeks, it would be more appropriate to evaluate the surface roughness based on the Rz values, as the measurements may be affected surface irregularities.

### 3.7. Morphological Examination of Fracture Surfaces

**Figure 11:**
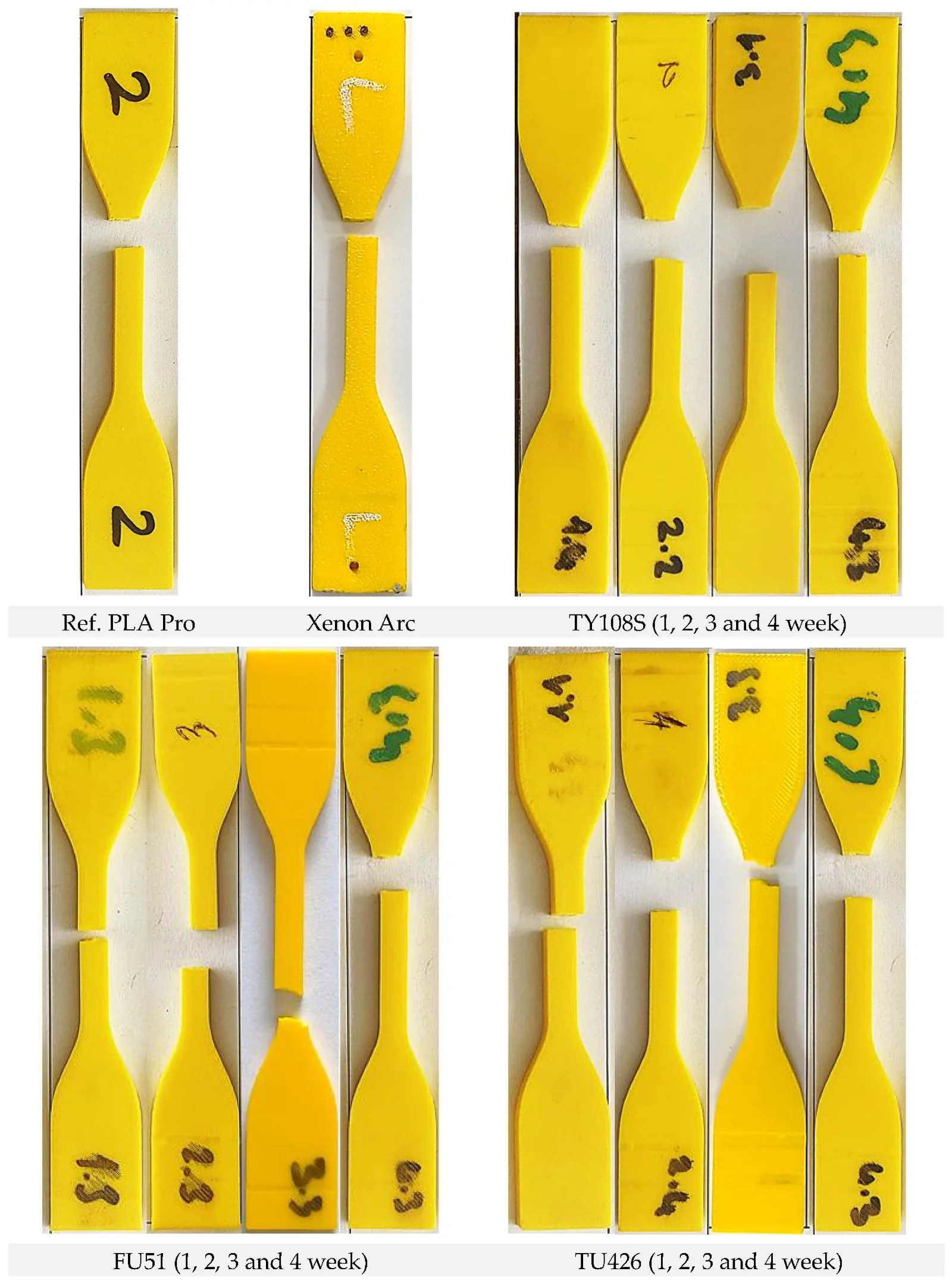
Fracture surfaces of the reference and aged specimens after the tensile test.

The images of the fracture surfaces of the specimens obtained after the tensile test are presented in Figure 11. Except for FU51 (Weeks 1 and 2) and TU426 (Week 1), all specimens fractured in the region below the gripping jaw. The fracture direction was perpendicular to the loading direction. Fracture occurring at the boundary of the gauge section or at the grip transition (radius) during tensile testing of 3D-printed parts is a frequently encountered phenomenon in the literature [46,47]. Many studies have reported that, depending on the layer orientation, fracture tends to occur in the grip transition region rather than in the gauge section, indicating anisotropy (directional dependence) arising from the nature of additive manufacturing [48,49,50].

**Figure 12:**
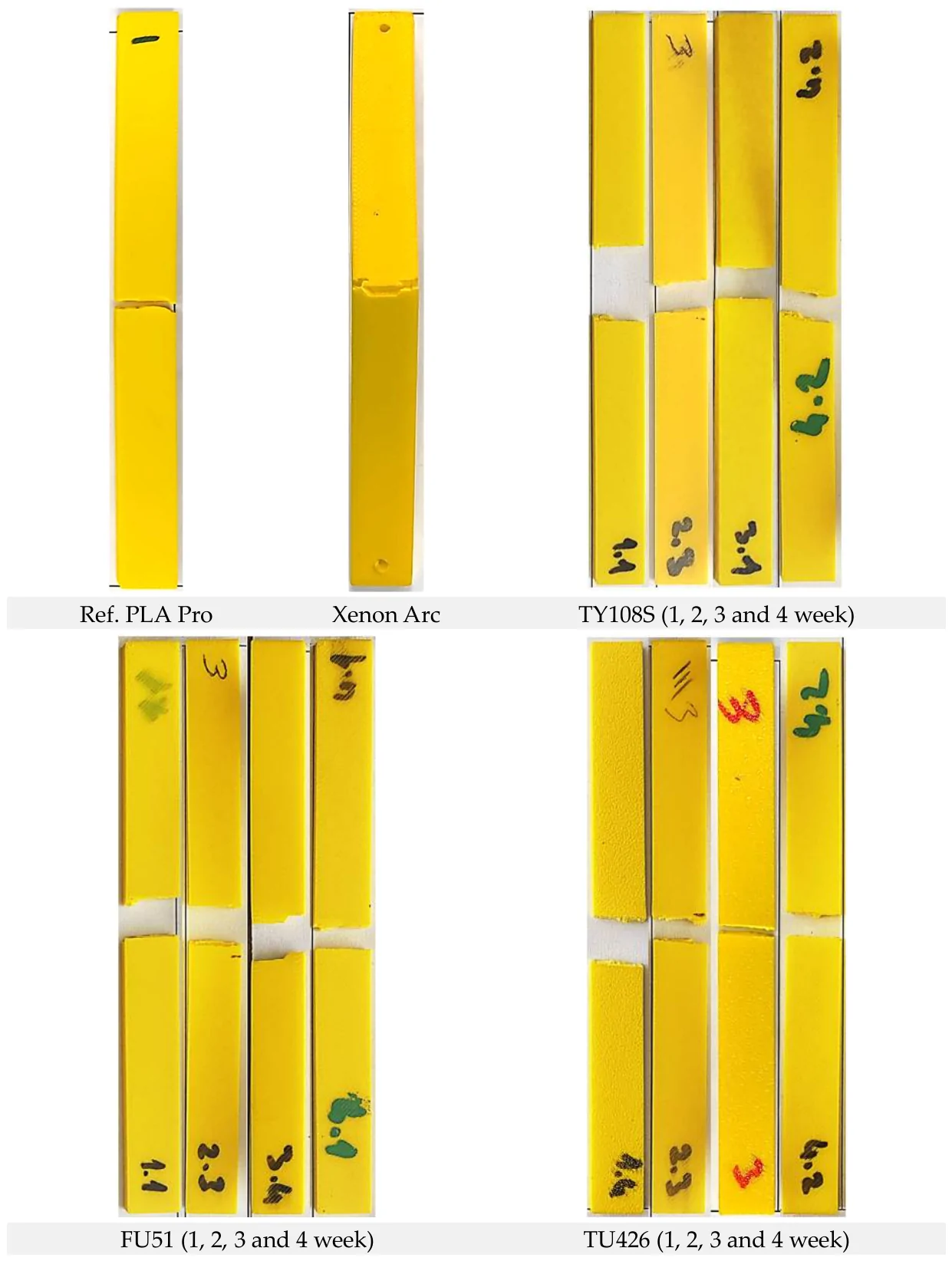
Fracture surfaces of the reference and aged specimens after the flexural test.

The images of the fracture surfaces of the specimens obtained after the flexural test are presented in Figure 12. Except for the specimens subjected to Xenon Arc aging, all specimens fractured into two separate pieces.

## 4. Conclusions

The ease of use and accessibility of 3D printers have rapidly expanded their application areas. One of the emerging application areas of FDM is the manufacturing industry. Conventional manufacturing of custom-made fixtures is costly and time-consuming. Therefore, 3D printers have increasingly been used to produce fixtures. In this study, 3D-printed specimens were exposed to three different coolants (TY108S, FU51, and TU426) for four different aging periods (1–4 weeks). In addition, accelerated aging was performed using a Xenon Arc aging chamber. The study aimed to investigate the potential of 3D-printed PLA Pro parts for manufacturing-related applications and to compare the effects of different aging conditions. When the tensile strength results were examined, an increase in strength was observed under all coolant and Xenon Arc aging conditions, except for FU51 at Week 1. The increase observed after Xenon Arc aging may be due to the effect of heat. Such a phenomenon could potentially be linked to the rearrangement of polymer macromolecules induced by prolonged exposure to elevated temperatures [51]. The mass increase of 0.18–0.95% observed after aging in the coolants indicates that the PLA Pro specimens absorbed components of the coolants. However, there is no direct correlation between mass increase and tensile strength.

Regarding the flexural force results, a decrease was observed particularly in the specimens immersed in FU51 and TU426 coolants at Week 3. In contrast, only a limited change was observed in the specimens exposed to TY108S. Xenon Arc aging resulted in a considerable increase in flexural force compared with the reference specimen.

When the optical properties of the specimens were evaluated, the highest total color change was observed after Xenon Arc aging. In contrast, the color changes in the specimens exposed to the coolants did not differ considerably from those of the reference specimens. Regarding surface gloss, higher changes were observed in the coolant-exposed specimens compared with the reference specimens.

After Xenon Arc aging, the surface roughness of the specimens decreased, whereas it increased in the specimens exposed to the coolants. In the manufacturing industry, surface roughness affects the quality of clamping. Up to a certain level, an increase in surface roughness may reduce the tendency for slipping by increasing mechanical interlocking and friction between the workpiece and the clamping surface. However, excessive surface roughness may prevent uniform contact over the entire surface. Therefore, the surface roughness of the parts should be kept under control. In conclusion, PLA and PLA-based materials can be used in the manufacturing industry due to their ease of printing, although the mechanical properties showed measurable changes under the investigated aging conditions.

Taken together, the tensile strength and flexural force results reveal a consistent pattern: (i) coolant type has a statistically significant main effect on both mechanical properties, with TY108S producing the highest numerical mean for both; (ii) the main effect of exposure duration (7–28 days) remains at the margin of significance for both properties without exceeding the 5% threshold; and (iii) 600 h of Xenon Arc aging is associated with a statistically non-significant change in UTS but a statistically significant increase in flexural force. The greater sensitivity of the flexural test to the stress distribution at the specimen surface compared with UTS, together with the tendency of Xenon-induced photochemical effects to concentrate primarily at the specimen surface [36,52], may partly explain this difference in sensitivity between the two properties.

The initial immersion experiments showed that the mass change of the PLA Pro specimens during the first 24 h remained relatively low, with maximum average increases of approximately 0.25%, 0.25%, and 0.30% for TY108S, FU51, and TU426, respectively.

In this study, a statistical analysis was conducted by combining two-way and one-way ANOVA with Tukey’s post hoc tests for both tensile strength and flexural force. The findings show that coolant type has a statistically significant main effect on both mechanical properties, whereas the effect of exposure duration remains at the margin of significance.

Overall, the results indicate that the investigated PLA Pro specimens maintained their mechanical performance under the specific coolant and accelerated aging conditions considered in this study, although measurable changes were observed in tensile strength, flexural force, hardness, surface roughness, gloss, and mass. These findings suggest that 3D-printed PLA Pro may have potential for manufacturing-related applications; however, the results should be interpreted within the limitations of the present study, which considered a single PLA Pro material (for yellow color filament only), one FDM processing condition, four weeks of coolant exposure, and 600 h of Xenon Arc exposure. It should also be noted that the mechanical property trends obtained from standard specimens cannot be directly generalized to complex 3D-printed structural components, as the mechanical response of such components may be strongly influenced by their geometry, internal structure, infill characteristics, layer orientation, and loading conditions [53]. Further studies involving dimensional stability, fatigue, creep, clamping performance, fixture loading, and long-term service life testing are required to assess the suitability of 3D-printed PLA Pro parts under actual manufacturing conditions.

## Figures and Tables

**Table 1:** Experimental setup of study.

Group–1		Group–2	
Coolant	Time (Week)	Accelerated aging	Time (Hour)
TY108S	1 2 3 4	Xenon Arc	600
FU51			
TU426			

**Table 2:** Stress–strain values of specimens exposed to coolants.

Time	Coolant					
	TY108S		FU51		TU426	
	Stress (MPa)	Strain (%)	Stress (MPa)	Strain (%)	Stress (MPa)	Strain (%)
Week 1	44.1 ± 1.8	7.0 ± 1.2	39.9 ± 1.7	9.1 ± 1.5	41.6 ± 1.7	8.6 ± 1.4
Week 2	48.4 ± 2.1	3.7 ± 0.7	47.1 ± 2.2	4.4 ± 0.9	42.8 ± 1.9	3.5 ± 0.7
Week 3	45.8 ± 1.9	4.1 ± 0.9	42.4 ± 1.9	3.9 ± 0.4	42.9 ± 2.1	3.9 ± 0.7
Week 4	47.7 ± 1.8	4.9 ± 1.1	43.4 ± 1.7	5.4 ± 1.1	46.4 ± 1.9	5.9 ± 1.1

**Table 3:** Two-way ANOVA results for the tensile strength (UTS) of the PLA Pro specimens (R^2^ = 80.32%; adjusted R^2^ = 63.91%).

Source of Variation	df	Adj SS	Adj MS	F	*p*
Coolant Type	2	27.20	13.598	5.31	0.047
Exposure Time (Week)	3	35.55	11.850	4.62	0.053
Error	6	15.38	2.563	—	—
Total	11	78.12	—	—	—

**Table 4:** Tukey HSD pairwise comparison results for coolant type (UTS).

Group 1	Group 2	Mean Diff. (MPa)	95% CI	*p* (adj.)
FU51	TU426	0.225	[−4.47; 4.92]	0.990
FU51	TY108S	3.300	[−1.40; 8.00]	0.177
TU426	TY108S	3.075	[−1.62; 7.77]	0.215

**Table 5:** One-way ANOVA results encompassing the reference, xenon arc-aged, and coolant-exposed groups (UTS).

Source of Variation	df	SS	MS	F	*p*
Between Groups (Ref/Xenon/TY108S/FU51/TU426)	4	43.06	10.764	1.90	0.194
Error	9	50.93	5.659	—	—
Total	13	93.99	—	—	—

**Table 6:** Force–displacement values of specimens exposed to coolants.

Time	Coolant					
	TY108S		FU51		TU426	
	Force (N)	Disp. (mm)	Force (N)	Disp. (mm)	Force (N)	Disp. (mm)
Week 1	135.8 ± 5.7	11.6 ± 2.2	130.6 ± 5.3	16.4 ± 2.4	130.3 ± 4.8	18.5 ± 2.5
Week 2	130.5 ± 5.1	14.6 ± 1.8	127.9 ± 4.8	16.8 ± 1.8	123.6 ± 4.7	17.6 ± 1.7
Week 3	132.0 ± 4.8	18.3 ± 2.3	118.2 ± 3.9	24.4 ± 2.1	123.6 ± 3.2	21.8 ± 1.8
Week 4	133.0 ± 4.9	14.3 ± 1.7	130.8 ± 4.6	19.9 ± 1.4	130.8 ± 3.8	19.8 ± 1.8

**Table 7:** Two-way ANOVA results for the flexural force of the PLA Pro specimens (R^2^ = 80.01%; adjusted R^2^ = 63.36%).

Source of Variation	df	Adj SS	Adj MS	F	*p*
Coolant Type	2	91.34	45.670	5.27	0.048
Exposure Time (Week)	3	116.96	38.988	4.50	0.056
Error	6	52.02	8.670	—	—
Total	11	260.32	—	—	—

**Table 8:** Tukey HSD pairwise comparison results for coolant type (flexural force).

Group 1	Group 2	Mean Diff. (N)	95% CI	*p* (adj.)
FU51	TU426	0.200	[−8.35; 8.75]	0.998
FU51	TY108S	5.950	[−2.60; 14.50]	0.183
TU426	TY108S	5.750	[−2.80; 14.30]	0.201

**Table 9:** One-way ANOVA results encompassing the reference, xenon arc-aged, and coolant-exposed groups (flexural force).

Source of Variation	df	SS	MS	F	*p*
Between Groups (Ref/Xenon Arc/TY108S/FU51/TU426)	4	443.47	110.867	5.90	0.013
Error	9	168.98	18.776	—	—
Total	13	612.45	—	—	—

**Table 10:** Tukey–Kramer pairwise comparison results encompassing the reference, xenon arc-aged, and coolant-exposed groups (flexural force).

Group 1	Group 2	Mean Diff. (N)	95% CI	*p* (adj.)
FU51	Ref.	5.025	[−11.27; 21.32]	0.832
FU51	TU426	0.200	[−10.10; 10.50]	1.000
FU51	TY108S	5.950	[−4.35; 16.25]	0.363
FU51	Xenon Arc	21.525	[5.23; 37.82]	0.011
Ref.	TU426	−4.825	[−21.12; 11.47]	0.851
Ref.	TY108S	0.925	[−15.37; 17.22]	1.000
Ref.	Xenon Arc	16.500	[−4.11; 37.11]	0.133
TU426	TY108S	5.750	[−4.55; 16.05]	0.392
TU426	Xenon Arc	21.325	[5.03; 37.62]	0.011
TY108S	Xenon Arc	15.575	[−0.72; 31.87]	0.062

**Table 11:** Color measurement test results.

Environmental Conditions	Time	L*	a*	b*	ΔE*
Reference	-	81.10	12.15	79.23	-
Xenon Arc	600 h	81.65	13.27	63.71	15.57
TY108 S	1. Week	81.00	12.08	79.35	0.17
	2. Week	81.13	12.10	79.49	0.26
	3. Week	81.21	12.00	79.63	0.44
	4. Week	81.15	11.93	79.97	0.77
FU51	1. Week	80.98	12.13	79.13	0.16
	2. Week	80.58	12.07	78.51	0.89
	3. Week	80.61	11.94	78.55	0.86
	4. Week	80.75	12.05	78.82	0.55
TU426	1. Week	80.93	12.16	79.36	0.21
	2. Week	80.88	12.02	79.57	0.42
	3. Week	80.26	11.80	77.98	1.54
	4. Week	80.96	12.02	79.58	0.40

**Table 12:** Gloss values of specimens.

Environmental Conditions	Time	20°	60°	85°
Reference	**-**	1.2	8.6	15.3
Xenon Arc	600 h	1.0	6.4	12.2
TY108S	1. Week	1.2	10.7	13.5
	2. Week	1.3	11.3	13.1
	3. Week	1.2	10.4	12.9
	4. Week	1.2	8.3	11.0
FU51	1. Week	1.4	10.1	12.2
	2. Week	1.4	10.3	11.4
	3. Week	1.1	10.6	10.5
	4. Week	1.3	10.2	10.4
TU426	1. Week	1.2	8.9	10.6
	2. Week	1.3	10.7	12.7
	3. Week	0.9	9.4	11.8
	4. Week	1.2	10.2	12.8

**Table 13:** Digital microscope images of the specimens before and after aging.

	Reference		Xenon Arc	
	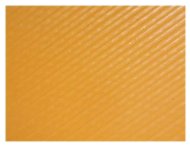		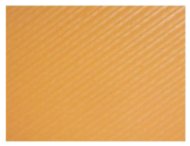	
Time	TY108S	FU51		TU426
Week 1	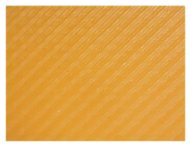	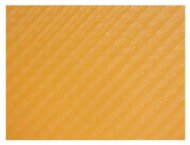		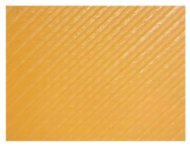
Week 2	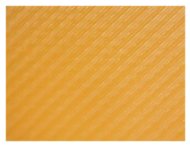	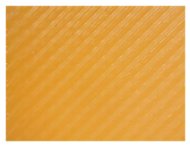		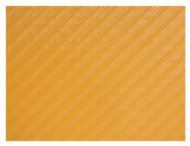
Week 3	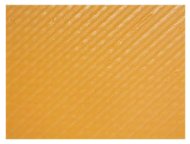	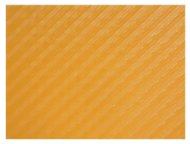		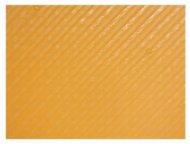
Week 4	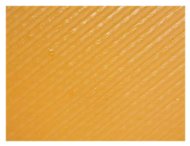	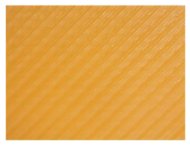		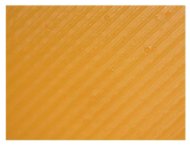

## Data Availability

The original contributions presented in this study are included in the article. Further inquiries can be directed to the corresponding author.
